# Exploration of mitochondrial defects in sarcopenic hip fracture patients

**DOI:** 10.1016/j.heliyon.2022.e11143

**Published:** 2022-10-19

**Authors:** Stefan Hintze, Lisa Baber, Fabian Hofmeister, Stefanie Jarmusch, Vanessa Todorow, Stefan Mehaffey, Fabiana Tanganelli, Uta Ferrari, Carl Neuerburg, Daniel Teupser, Martin Bidlingmaier, Jair Gonzalez Marques, Berthold Koletzko, Benedikt Schoser, Michael Drey, Peter Meinke

**Affiliations:** a Friedrich-Baur-Institute at the Department of Neurology, LMU Klinikum, LMU Munich, Germany; b Department of Medicine IV, Geriatrics, LMU Klinikum, LMU Munich, Germany; c Department of General-, Trauma- and Reconstructive Surgery, LMU Klinikum, LMU Munich, Germany; d Institute of Laboratory Medicine, LMU Klinikum, LMU Munich, Germany; e Department of Medicine IV, Endocrinology, LMU Klinikum, LMU Munich, Germany; f Department Pediatrics, Dr. von Hauner Children's Hospital, LMU Klinikum, LMU Munich, Germany

**Keywords:** Sarcopenia, Tricarboxylic acid cycle, TCA, Mitochondria, Metabolism, Myoblast culture

## Abstract

Severe cases of age-related loss of muscle function and mass are clinically unique to sarcopenia. Mitochondrial dysfunction has been associated with aging and sarcopenia, but the causal connection in this context is not well eluded. Here we investigated different aspects of mitochondrial respiration in sarcopenia. Open muscle biopsies were taken from a total of 31 hip fracture patients, older than 70 years. Patients were assigned a sarcopenia Z-score based on EWGSOP2 criteria. Primary myoblast cultures were generated from the muscle tissue samples and used for real time metabolic measurement. Muscle and serum samples showed correlation of high Z-scores with reduced mitochondrial complex I activity, increased tricarboxylic acid cycle (TCA) metabolites, reduced vitamin D_3_ levels, and signs of an altered iron metabolism. Primary myoblast cultures gained from the same muscle biopsies did not show significant mitochondrial defects. We hypothesize that a sum of external consequences, including vitamin D_3_ deficiency and iron deficiency caused by disturbances in the iron metabolism, result in complex I deficiency, which in turn affects the TCA and contributes to muscle weakness and loss.

## 1 Introduction

Skeletal muscle forms with ∼30–40% of body mass typically the largest portion of the human body (Kim et al., 2002). During aging it comes to a decline of muscle mass, which usually begins between the 3rd and 4th decade and accelerates around the 6th to 8th decade (Janssen et al., 2000; Lexell et al., 1988; Silva et al., 2010). While this appears to be a slow but normal process during aging, it emanates to a more rapid loss of muscle mass in some elder individuals. Clinically, this is a geriatric syndrome defined as sarcopenia, an age related progressive loss of muscle function and mass (Cruz-Jentoft et al., 2010, 2019; Fielding et al., 2011). Sarcopenia is associated with several increased risks including physical disability, dysmobility, increased risk of falls and fractures, and loss of independency (Beaudart et al., 2017; Yeung et al., 2019). The co-occurrence of several aspects can be seen especially when it comes to falls. Apart from reduced gait stability, caused by loss of muscle mass and function, patients often have additional osteoporosis, which consequently results in an increased risk of hip fractures (Cawthon et al., 2015; Hida et al., 2013; Landi et al., 2012; Oliveira and Vaz, 2015; Yeung et al., 2019). This co-occurrence of sarcopenia and osteoporosis, resulting in worse outcomes than any condition alone, has even been described as a separate geriatric syndrome, called osteosarcopenia (Huo et al., 2015). Sarcopenic patients do also have an increased risk of shoulder pain (Han et al., 2021) and sarcopenia is likely to be associated with poorer quality of life after lumbar surgeries (Wu et al., 2021).

Between 5% and 13% of people between 60 and 70 are reported to suffer from sarcopenia, but in people older than 80 years it's already 11–50% (von Haehling et al., 2010). Considering demographic changes and increasing life expectancy, the total number of people suffering from sarcopenia is likely to increase over the coming years, thus presenting a massive burden to healthcare systems.

There are many known factors contributing to the development of sarcopenia, some of them are lack of activity, malnutrition, anorexia, inflammation, hypovitaminosis D, reduced blood circulation, as well as genetic and epigenetic predisposal (Stangl et al., 2019). Changes in metabolism of aging skeletal muscle are one factor that can be highlighted (Rezuş et al., 2020).

Mitochondrial dysfunction has been associated with aging (Ferri et al., 2020). In fact it is considered to be one of the hallmarks of aging (López-Otín et al., 2013). Mitochondrial disturbances described in aging include an increased frequency of mitochondrial DNA mutations (Lee et al., 1997; McKenzie et al., 2002), decreased synthesis and activity of mitochondrial proteins (Rooyackers et al., 1996), altered mitochondrial dynamics (Romanello, 2020), and increased generation of reactive oxygen species (Mansouri et al., 2006). Despite this wealth of information it is still unclear if mitochondrial dysfunction is causing aging, or happening as a result of aging (Hipkiss, 2010). The answer to this question could be of import when it comes to the development of therapies for sarcopenia.

In this study, we analyzed different aspects of mitochondrial respiration. Sarcopenia severity was described by a sarcopenia Z-score based on EWGSOP2 criteria in elderly patients with hip fracture. Biopsies from these patients, as well as myoblast cultures generated from those biopsies, were analyzed regarding mitochondrial function. In addition, we analyzed metabolites in serum gained from the patients.

## 2 Patients and methods

### 2.1 Participants and diagnosis of sarcopenia

The patient cohort used in this study included 31 participants, aged 70 years and older, who underwent operative treatment due to a proximal femur fracture. Exclusion criteria were age: < 70 years, the presence or evidence of hereditary muscular and neuromuscular diseases (myasthenia gravis, muscular dystrophy, ALS, polio), severe dementia, chronic inflammatory diseases (e.g. Crohn's disease, ulcerative colitis, rheumatoid arthritis, systemic corticosteroid therapy) and cancer therapy in the last 5 years. Patients were recruited between November 2017 and March 2019 at the LMU university hospitals, Munich. The study protocol was approved by the Ethics Committee of the Medical Faculty of LMU Munich (IRB-No. 328-15) and has been performed in accordance with the ethical standards laid down in the 1964 Declaration of Helsinki and its later amendments.

For a metric measure of the degree of sarcopenia, a sarcopenia Z-score was calculated based on EWGSOP2 criteria, using the individual participant's data and the standard deviation (SD) obtained from the baseline data of the study cohort (Kemmler et al., 2016). Following formulae were used depending on the sex of the individuum:

*Z-Score (men) = ((27kg − individual handgrip strength)/SD handgrip strength) + ((7kg/m^2^ − individual SMI)/SD SMI)*
*Z-Score (women) = ((16kg − individual handgrip strength)/SD handgrip strength) + ((5.5kg/m^2^ − individual SMI)/SD SMI)*

We used a Saehan DHD-1 Digital Hand Dynamometer, with the patient lying supine to assess handgrip strength based on the maximal value of three consecutive measurements of both hands. The skeletal muscle index [SMI, (kg/m^2^)] was estimated by dividing appendicular lean mass (aLM) by squared body height. To calculate the aLM we used the equation developed by Sergi et al. (2015). Bioelectrical impedance analysis (BIA) was performed using the SFB7 (ImpediMed®). For the measurement patients were put in supine position, with surface electrodes placed on the wrist and ankle contralateral to the side of the fracture.

### 2.2 Muscle biopsies and cell culture

During hip fracture surgery open biopsies of the vastus lateralis muscle were obtained. Muscle specimens were taken in the area of insertion of the vastus lateralis muscle close to the greater trochanter. These biopsies were divided under sterile conditions in two parts: one part, which was used for cryo-conservation, was shock-frozen in liquid nitrogen and stored at −80 °C. The second part, used for tissue culture, was initially stored in Solution A (HEPES 7.2 g/l, sodium chloride 7.6 g/l, potassium chloride 0.224 g/l, glucose 1.98 g/l, phenol red 1.134 × 10^−4^ g/l, pH 7.4) and later chopped into 1 mm pieces. These pieces were stirred vigorously in a solution containing Solution A and Trypsin-EDTA on a magnetic stirrer for 15 min. The resulting cell suspension was washed with DMEM (supplemented with 10% FCS and 40 μg/ml gentamycin), and cells were pelleted by centrifugation. The cell pellet was resuspended and cultured in skeletal growth medium (PeloBiotech), supplemented with 5 ml GlutaMAX™ (Gibco) and 2 ml penicillin, at 37 °C in 5% CO_2_ atmosphere. Cells were kept from reaching confluence to avoid differentiation.

### 2.3 Real time metabolic measurements

Metabolic measurements on primary human myoblast cultures were performed following the second passage using the Seahorse XFp Extracellular Flux Analyzer (Seahorse Bioscience). For this, myoblasts were seeded in XFp Cell Culture Miniplates (103025-100, Seahorse Bioscience) at a density of 1.5 × 10^4^ cells per well. Cell density was assessed using an automated cell counter (TC20, BioRad). Mitochondrial function respectively glycolytic activity was measured using the Mito Stress Test Kit (Agilent) and the Glycolysis Stress Test Kit (Agilent) according to the manufacturer's instructions. At least three measurements were done per sample and test and the data were normalized to the number of cells and analyzed for each well.

### 2.4 Respiratory chain activity

To assess the respiratory chain activity cryoconserved muscle samples were used. Respiratory chain complex measurements were performed as published by others (Fischer et al., 1986).

### 2.5 Serum analysis

Blood samples were obtained on the third postoperative day. After centrifugation, the serum was stored at −80 °C until analysis.

A clinical chemistry analysis for iron was performed with photometric assays. Transferrin and sTfR (soluble transferrin receptor) were analyzed with immunoturbidimetric assays, ferritin was analyzed by a particle enhanced immunological turbidimetry. All clinical chemistry parameters were measured on a cobas 8000 automated analyzer (Roche, Rotkreuz, Switzerland).

For the quantification of tricarboxylic acid cycle (TCA) intermediates in serum, measurements were performed using high performance liquid chromatography (1200, Agilent, Waldbronn, Germany) coupled to a tandem mass spectrometer (4000QTRAP, Sciex, Darmstadt, Germany) (HPLC-MS/MS) in negative ion mode using electrospray ionization (ESI) as previously described (Marchioro et al., 2020). Briefly, 50 μL of serum were precipitated with 450 μL methanol and filtrated. One hundred μL of the supernatant were dried under N_2_ stream and reconstituted in 50 μL of H_2_O. Chromatographic separation was achieved by injecting 5 μL of sample into a Kinetex F5 core-shell HPLC column, 150 × 2.1 mm, 2.6 μm particle size (Kinetex F5, Phenomenex, Aschaffenburg, Germany). A gradient elution flow rate of 250 μL/min–using H_2_O (1% formic acid) as mobile phase A and methanol/isopropanol (50/50) with 1% formic acid as mobile phase B–was applied starting with 1% of B held for 1 min, increasing to 65% B for 6 min, and returning to initial conditions in 0.5 min.

Serum 25-hydroxy (OH) vitamin D (25OHD) concentrations were measured on the IDS-iSYS multidiscipline automated analyzer (Immunodiagnostic Systems Limited, Boldon, UK).

### 2.6 Statistical analysis

Group differences (non-sarcopenia versus probable-sarcopenia and sarcopenia) were calculated by ANOVA. The statistical significance was set at *P* < 0.05. Correlation (*R*) and significance (*P*) for the scatter plots were calculated using the R package ggpmisc.

## 3 Results

### 3.1 General participant cohort

We generated vital myoblast cultures from 31 patients (17 female, 14 male). The average age of the female patients was 81.7 years (±5.8), that of the male patients 82.7 years (±6.5). Five patients (2 male and 3 female) were considered sarcopenic (skeletal muscle index [SMI (kg/m^2^)] < 7 (♂)/< 5.5 (♀) and a handgrip strength <27 kg (♂)/< 16 kg (♀)), 10 (7 male and 3 female) as probably sarcopenic (SMI >7 (♂)/> 5.5 (♀) but handgrip strength <27 kg (♂)/< 16 kg (♀)), and 16 (5 males and 11 females) non-sarcopenic (Table 1).

**Table 1:** General characteristics of the patient cohort (mean values and standard deviation).

	Total (n = 31)	Men (n = 14)			Women (n = 17)		
		Non- sarcopenic (n = 5)	Probable- sarcopenic (n = 7)	Sarcopenic (n = 2)	Non- sarcopenic (n = 11)	Probable- sarcopenic (n = 3)	Sarcopenic (n = 3)
**Age [y]**	82 ± 6	82 ± 6	82 ± 7	86 ± 1	81 ± 7	86 ± 5	80 ± 6
**Body height [cm]**		178 ± 11	175 ± 7	169 ± 13	163 ± 4	157 ± 3	169 ± 1
**Body weight [kg]**		80 ± 12	78 ± 11	49 ± 12	72 ± 11	64 ± 9	55 ± 5
**BMI [kg/m**^**2**^**]**		25.1 ± 1.2	25.4 ± 2.7	17.5 ± 7.0	27.0 ± 3.4	25.9 ± 3.0	19.2 ± 1.5
**Handgrip strength [kg]**		37.6 ± 5.8	22.5 ± 3.9	16.4 ± 4.2	23.0 ± 5.3	12.0 ± 2.7	13.4 ± 2.4
**SMI [kg/m**^**2**^**]**		8.0 ± 1.1	7.5 ± 0.6	5.5 ± 1.5	6.7 ± 0.9	6.8 ± 1.3	5.0 ± 0.5
**Z-score**		−2.0 ± 1.3	0.0 ± 0.7	2.4 ± 1.2	−2.2 ± 1.3	−0.6 ± 0.7	0.9 ± 0.3

### 3.2 Respiratory chain enzyme activity

**Figure 1:**
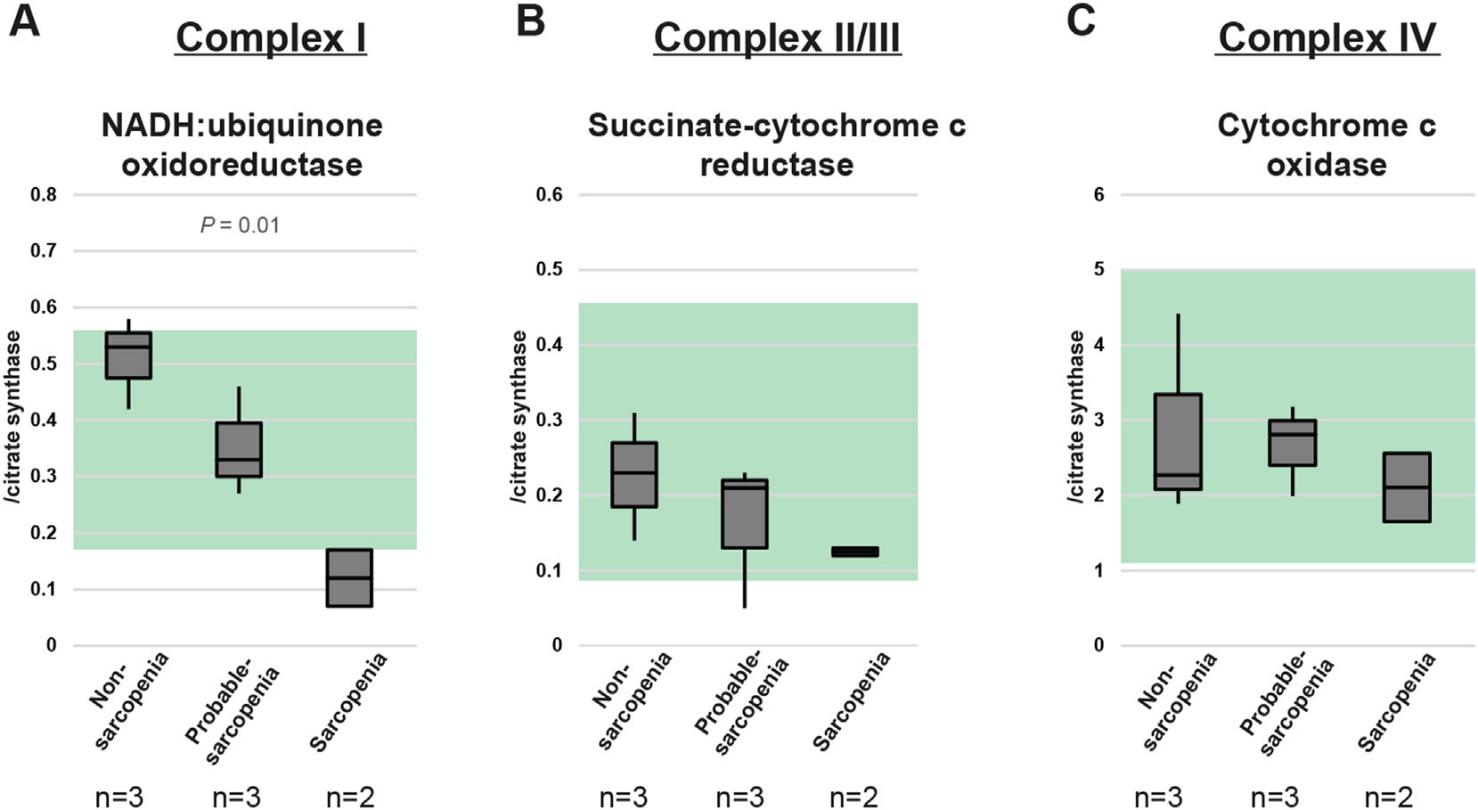
Activity of respiratory chain complexes in muscle. The activity (normalized to citrate synthase) of (A) complex I, (B) complex II/III and (C) complex IV is shown for non-sarcopenic (n = 3), probably-sarcopenic (n = 3), and sarcopenic patients (n = 2). Green areas are representing the reference range.

Muscle biopsies were used to assess the activity of the respiratory chain enzymes. Material was available for 3 non-sarcopenic, 3 probable sarcopenic, and 2 sarcopenic patients. To assess the complex I activity, we tested the activity of NADH dehydrogenase. Values were normalized to citrate synthase. Non-sarcopenic and probable-sarcopenic samples were within the reference range, although the activity in the probable-sarcopenic samples was reduced in comparison to the non-sarcopenic ones. NADH dehydrogenase activity in sarcopenic samples was reduced even further and below the reference (Figure 1a). The enzyme activity of complexes II and III was assessed via succinate-cytochrome c reductase activity and normalized to citrate synthase. Although the activity in probable-sarcopenia was lower than in non-sarcopenia and even further reduced in sarcopenia, the majority of all groups was within the reference range and the differences were smaller than for complex I (Figure 1b). Complex IV activity was determined by testing cytochrome c oxidase activity and normalization to citrate synthase. Here all groups had activities of a similar level and within the reference range (Figure 1c).

### 3.3 Real time metabolic measurements

As the measurement of the respiratory chain activity from muscle biopsies indicated disturbances in mitochondrial function, we used the myoblast cultures gained from muscle biopsies to investigate if this effect could be observed also in tissue culture. For this we used a total of 16 non-sarcopenic, 10 probable sarcopenic, and 5 sarcopenic primary myoblast cultures.

**Figure 2:**
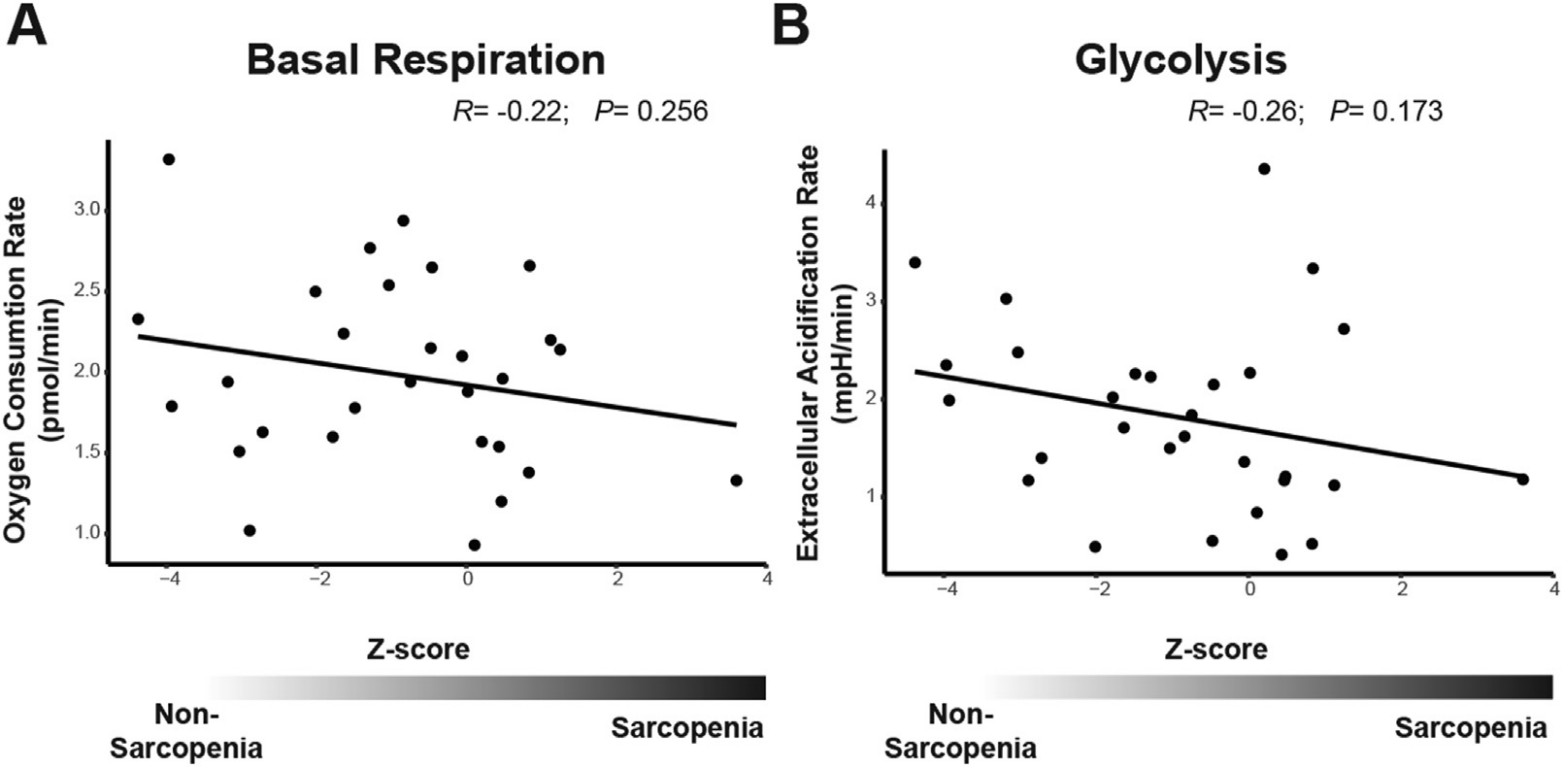
Metabolic measurement in 31 patient myoblast cultures: (A) basal respiration and (B) glycolysis plotted against the sarcopenia Z-score.

Investigating the mitochondrial function, we found a non-significant tendency towards lower basal respiration in myoblasts cultures from patients with higher Z-sores (Figure 2A).

Complementary to the mitochondrial function we investigated the glycolytic activity in the primary myoblast cultures. Glycolysis was also having a non-significant tendency towards lower values in myoblasts cultures from patients with higher Z-sores (Figure 2B). We could not identify any separate correlation with handgrip strength or skeletal muscle mass alone (Supplemental Figure S1).

### 3.4 Serum analysis

**Figure 3:**
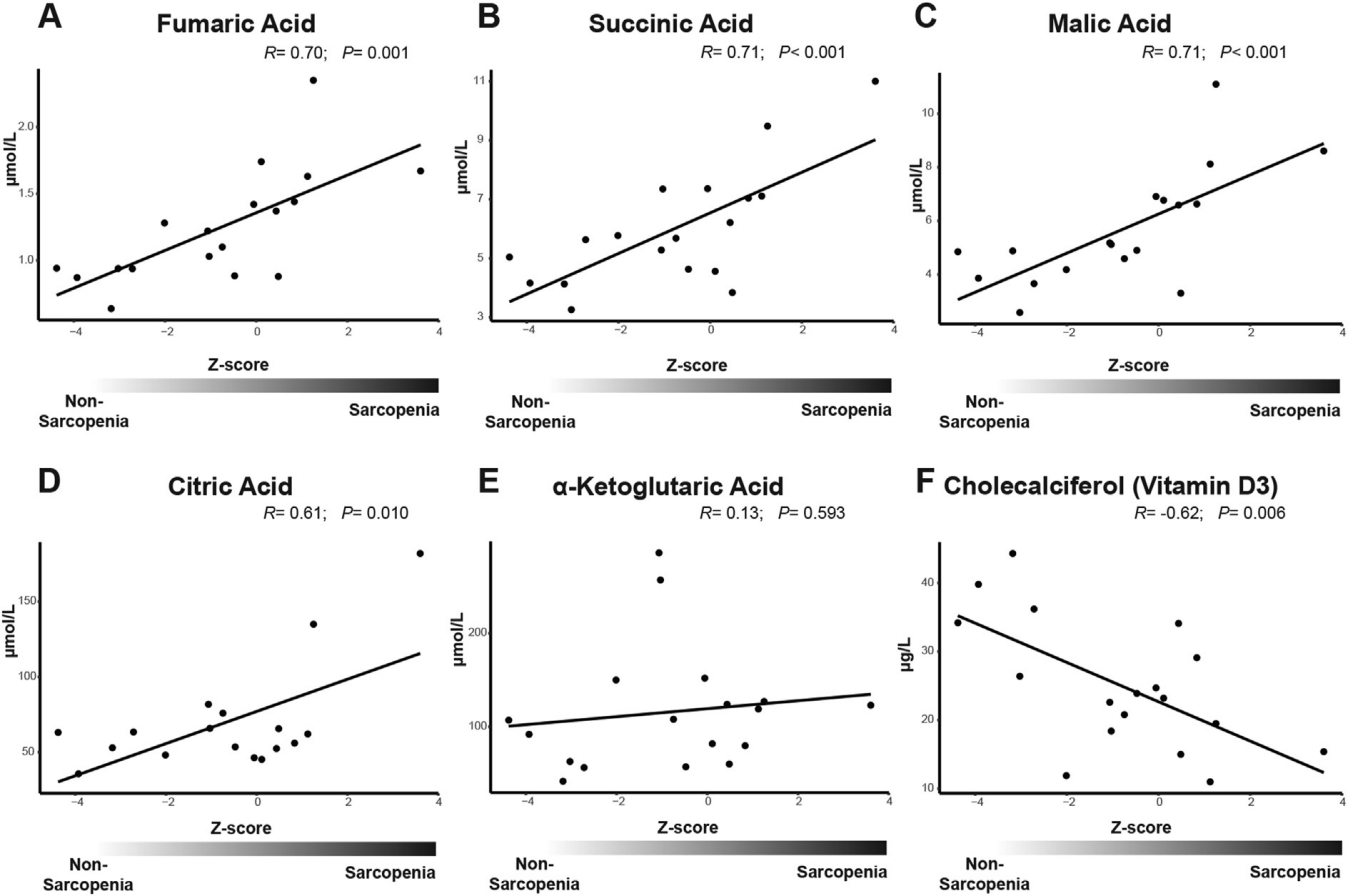
Serum analysis. The concentration of (A) fumaric acid, (B) succinic acid, (C) malic acid, (D) citric acid, (E) α-ketoglutaric acid, and (F) cholecalciferol (vitamin D_3_) measured in serum from 18 patients plotted against the sarcopenia Z-score.

Blood serum was available from 10 non-sarcopenic, 5 probable sarcopenic, and 3 sarcopenic patients. We found significant correlation between Z-score and serum concentrations of fumaric acid, succinic acid, malic acid, and citric acid. All were having increased serum concentrations in patients with high Z-scores (Figure 3A, B, C, D). The same tendency could be observed for α-ketoglutaric acid, although no significance could be determined (Figure 3E).

**Figure 4:**
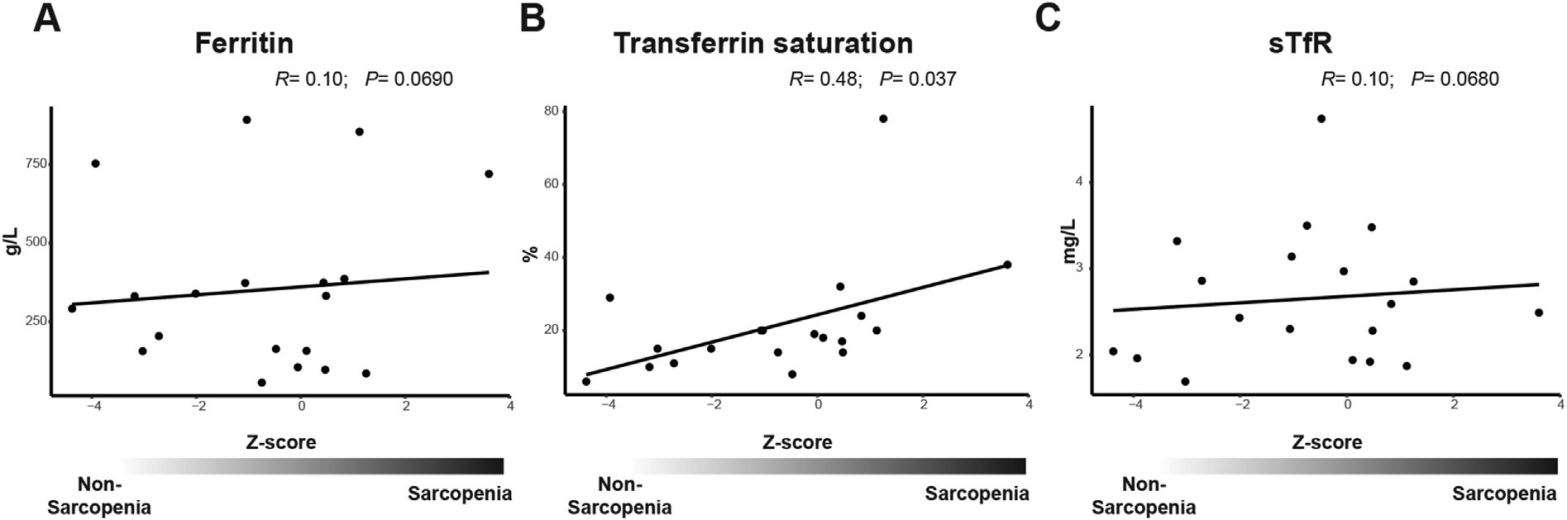
Iron status in serum. The concentration of (A) ferritin, (B) transferrin saturation and the (C) concentration of soluble transferrin receptor in a subset of 24 patients plotted against the sarcopenia Z-score.

We also considered additional factors that could impact on mitochondrial activity. A possible candidate is vitamin D, which levels did correlate significantly with the Z-score: the higher the Z-score the lower vitamin D serum levels (Figure 3F). Another factor that could impact on mitochondrial function is the iron status. For this serum-ferritin, transferrin saturation, and the soluble transferrin receptor were measured. Despite the same trend for all of them we found a significant correlation only for transferrin saturation (Figure 4A, B, C). For all factors handgrip strength and skeletal muscle mass separately behaved similar, indicating both are equally relevant for observed correlations (Supplemental Figures S2 and S3).

## 4 Discussion

Sarcopenia is defined as a loss of muscle mass and function (Cruz-Jentoft et al., 2010, 2019; Fielding et al., 2011). To allow for the fact that this disease is developing with age and thus there are many degrees of sarcopenia we did assign a sarcopenia Z-score to patients (Kemmler et al., 2016). This is supposed to reflect on the continuum of sarcopenia development.

We identified significantly reduced levels of respiratory chain complex I activity in muscle biopsies from sarcopenic patients. Probable-sarcopenic patients, which we consider to be pre-sarcopenic, had intermediate activities between sarcopenic and non-sarcopenic patients. This makes a correlation between complex I activity and the degree of sarcopenia likely. This is in agreement with other studies that reported complex I activity before in sarcopenic patients (Migliavacca et al., 2019) or a model organism for aging (Brunetti et al., 2020), although other studies found normal complex I activity in sarcopenic patients (Shah et al., 2009). Activities of complex II/III follow the same trend (similar to other published data (Migliavacca et al., 2019)), but the differences between the groups are less pronounced and already absent for complex IV activity. Thus, we conclude that the effect observed is not a general loss of mitochondria, but relatively specific for complex I. We also conclude that the differences in fiber type composition between sarcopenic and non-sarcopenic patients (Tanganelli et al., 2021) is not impacting on the result as we would expect a general reduction of all complexes if this would be the case.

Next, we wanted to test if primary human myoblast cultures show the same defect, which could make them a useful model system for sarcopenia. Due to the advanced age of the donors, myoblasts cultures were used for real time metabolic measurements in the second passage to avoid senescence of the cultures. Although there was a trend for decreased basal respiration in myoblast cultures from patients with high sarcopenia Z-scores, this was not statistically significant. We conclude that the defect causing the complex I deficiency in sarcopenic patients is not caused by genetic alterations in satellite cells, and thus likely due to external factors. Therefore, primary myoblast cultures gained from sarcopenic patients are limited in serving as a model for the *in vivo* situation.

**Figure 5:**
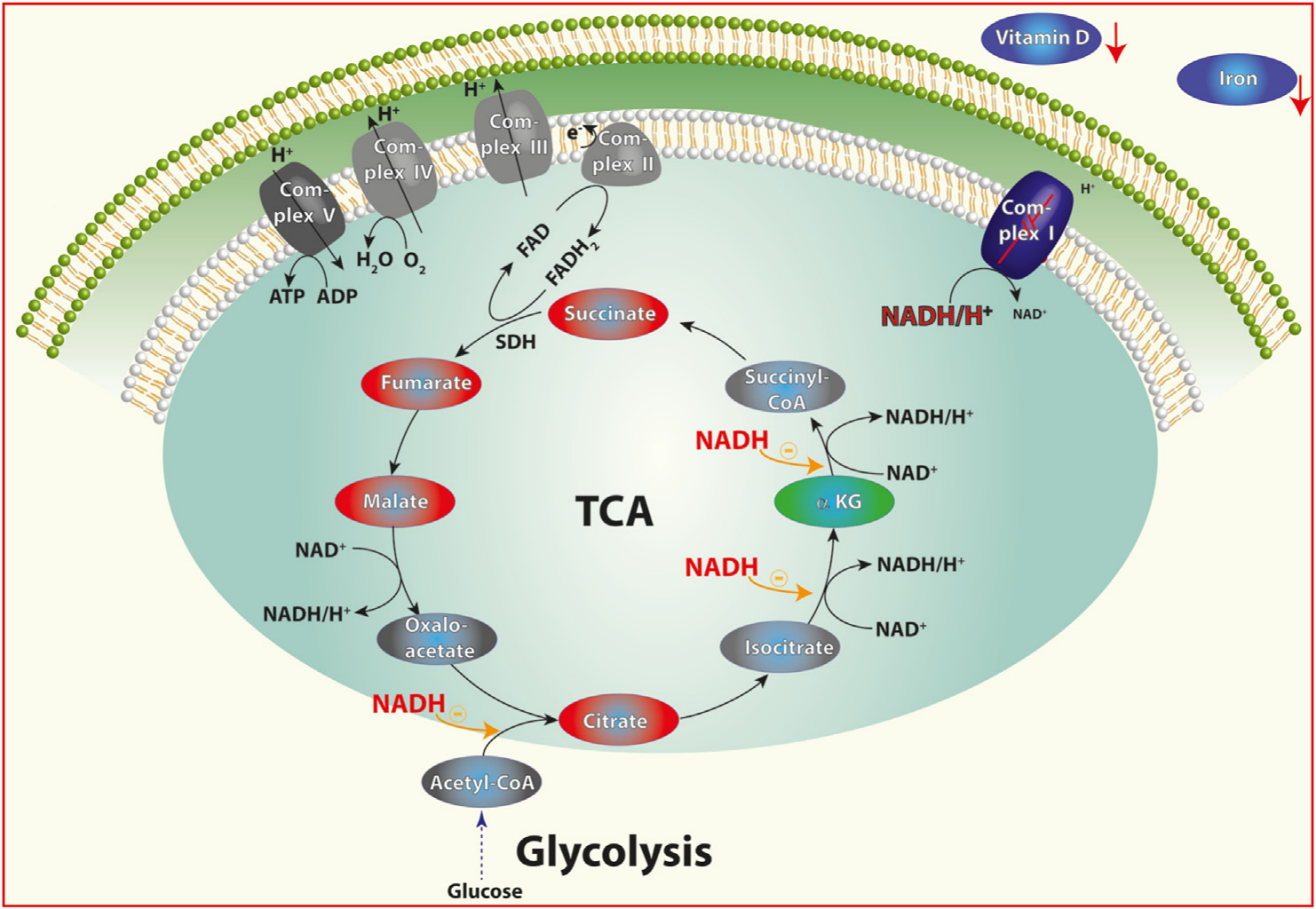
Schematic of the suggested mechanism.

We performed metabolic analyses from sera of our patient cohort to gain more information on additional predictive factors. We identified a striking increase of serum concentration of several metabolites of the tricarboxylic acid cycle (TCA) in patients with high Z-scores. Despite coming from a different tissue, these data might add information on the muscle pathology. The citric acids cycle produces NADH within mitochondria, which is then degraded to NAD by complex I (Sharma et al., 2009). A deficiency of complex I indicates reduced NADH degradation, which in turn has been shown to inhibit the TCA progression (Martínez-Reyes and Chandel, 2020). This would explain the increased levels of succinic acid, fumaric acid, and malic acid, all of which precede the reaction of malic acid to oxaloacetate in the TCA cycle that produces NADH. Thus, a complex I deficiency would result in NADH accumulation, which inhibits the progression of the TCA (Figure 5). Support for this hypothesis comes from a study showing reduced NAD levels in sarcopenia (Migliavacca et al., 2019), which fits with a reduced NADH degradation by complex I. This conclusion is based on the assumption that serum values reflect the metabolic state in skeletal muscle. We consider this likely in line with our conclusion that external factors (likely affecting the whole organism) are involved in the development of the complex I deficiency in sarcopenic patients.

A further contributor seems to be vitamin D_3_ (cholecalciferol) which is found at low levels in patients with high Z-scores and has been shown to play an important role in the mitochondrial metabolism (Montenegro et al., 2019). It also seems possible that disturbances in the iron metabolism contribute to the complex I deficiency. The respiratory complex I has been described to contain eight iron-sulfur clusters (Brandt, 2006; Sheftel et al., 2009), thus it appears possible that the amount of accessible iron could influence its activity. Indeed, it has been shown that iron deficiency can result in complex I deficiency in mouse and rat models (Bastian et al., 2020; Rineau et al., 2018). Further evidence for the import of iron regulation comes from a study showing complex I inhibition and following induced cell death by misregulated iron (Urrutia et al., 2017). The increased transferrin saturation with nearly unchanged levels of soluble transferrin receptor could indicate a disturbed iron metabolism resulting in iron deficiency and in turn in reduced complex I activity.

In summary, we hypothesize that extrinsic effects including vitamin D deficiency and iron deficiency, caused by disturbances in iron metabolism, result in complex I deficiency, which in turn inhibits the TCA and contributes to muscle weakness and fiber loss in sarcopenia. While this may hold true only in a subset of sarcopenic patients, it would allow for a targeted therapy (for example substitution with iron and vitamin D) that could be guided by assessment of TCA metabolites in blood serum. Thus, we present for the first time indications that reduced mitochondrial activity in sarcopenia is possible affected by malnutrition.

## 5 Limitations

In interpreting the present findings, some limitations should be considered. These are foremost the descriptive nature of this study and the relatively small sample size. It is important to mention that handgrip strength and SMI measurements were taken after hip fracture and surgery. Even though BIA is a technique to estimate the lean body mass, it is not considered as a gold standard for the quantification of muscle mass such as DXA is.

## Declarations

### Author contribution statement

Stefan Hintze: Performed the experiments; Analyzed and interpreted the data; Wrote the paper.

Lisa Baber: Performed the experiments; Analyzed and interpreted the data.

Fabian Hofmeister, Stefanie Jarmusch, Stefan Mehaffey, Fabiana Tanganelli, Carl Neuerburg and Berthold Koletzko: Contributed reagents, materials, analysis tools or data.

Vanessa Todorow and Uta Ferrari: Analyzed and interpreted the data.

Daniel Teupser, Martin Bidlingmaier and Jair Gonzalez Marques: Performed the experiments.

Benedikt Schoser: Conceived and designed the experiments.

Michael Drey and Peter Meinke: Conceived and designed the experiments; Wrote the paper.

### Funding statement

This study was supported by the EU project Keep Control, funded under the European Union’s Horizon 2020 research and innovation programmed under the Marie Skłodowska-Curie grant agreement No [721577]. The work of Berthold Koletzko and Jair Gonzalez Marques is supported by the European Commission H2020 Programme Lifecycle-733206, the European Joint Programming Initiative Projects NutriPROGRAM, and the German Research Council DFG (INST 409/224-1 FUGG), Berthold Koletzko is the Else Kröner Seniorprofessor of Paediatrics at LMU – University of Munich, financially supported by the Else Kröner-Fresenius-Foundation, the LMU Medical Faculty and the LMU University Hospitals. Peter Meinke is funded by the Deutsche Forschungsgemeinschaft (DFG, German Research Foundation) – Projektnummer [470092532].

### Data availability statement

Data included in article/supp. material/referenced in article.

### Declaration of interests statement

The authors declare no conflict of interest.

### Additional information

Supplementary content related to this article has been published online at [URL].
