# Supplementary material for: Exploration of mitochondrial defects in sarcopenic hip fracture patients
Source: Heliyon. 2022 Oct 19;8(10):e11143. doi: 10.1016/j.heliyon.2022.e11143 (PMC9593198; doi:10.1016/j.heliyon.2022.e11143)
Supplement: Figure S1 [file mmc1.docx]

**Max. Grip Strength SMI**


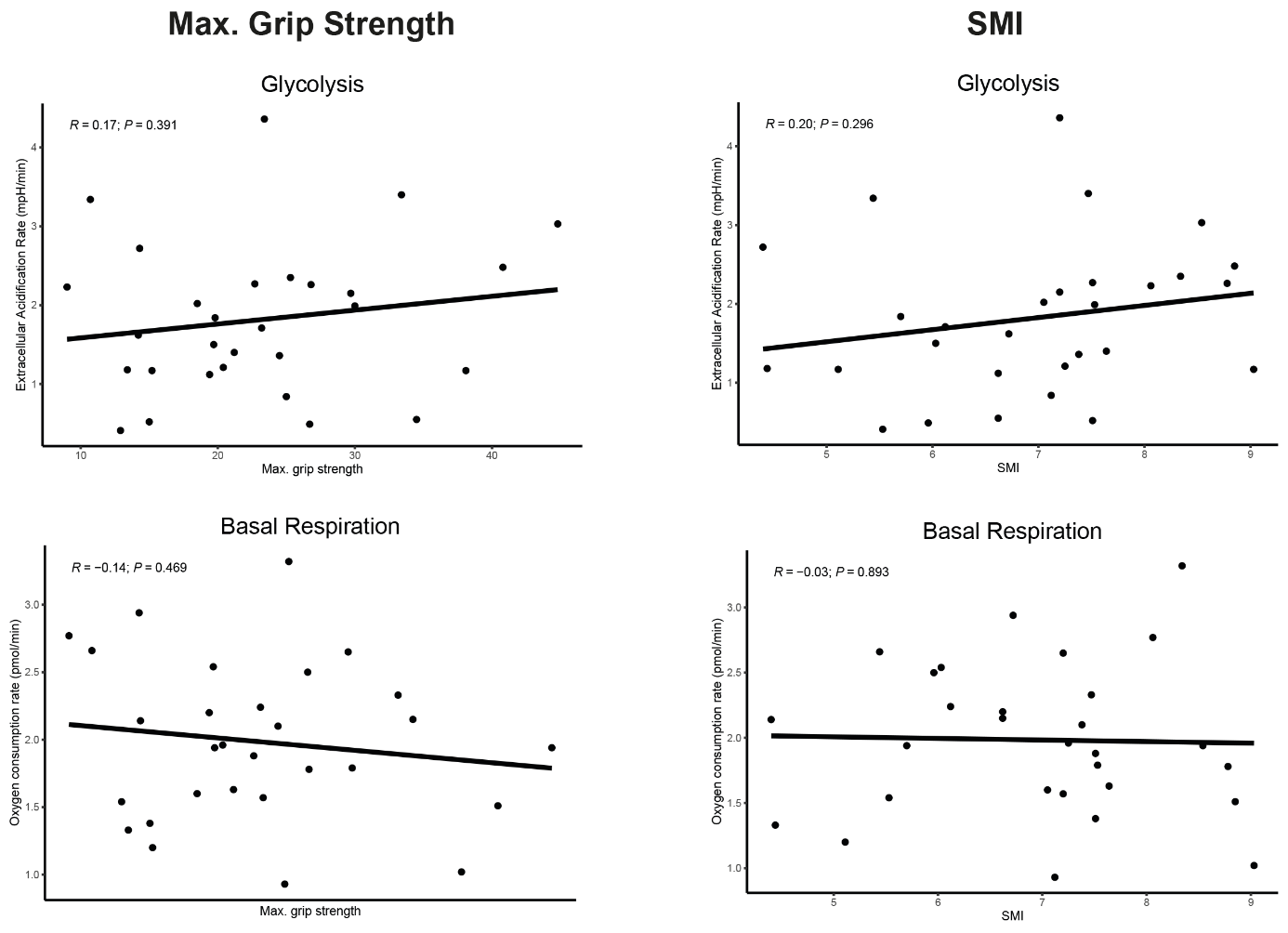


**Figure S1:** Metabolic measurement in 31 patient myoblast cultures: basal respiration and glycolysis plotted separately against handgrip strength (max. grip strength) and skeletal muscle mass (SMI).
