# Supplementary material for: Exploration of mitochondrial defects in sarcopenic hip fracture patients
Source: Heliyon. 2022 Oct 19;8(10):e11143. doi: 10.1016/j.heliyon.2022.e11143 (PMC9593198; doi:10.1016/j.heliyon.2022.e11143)
Supplement: Figure S2 [file mmc2.docx]

**Max. Grip Strength SMI**


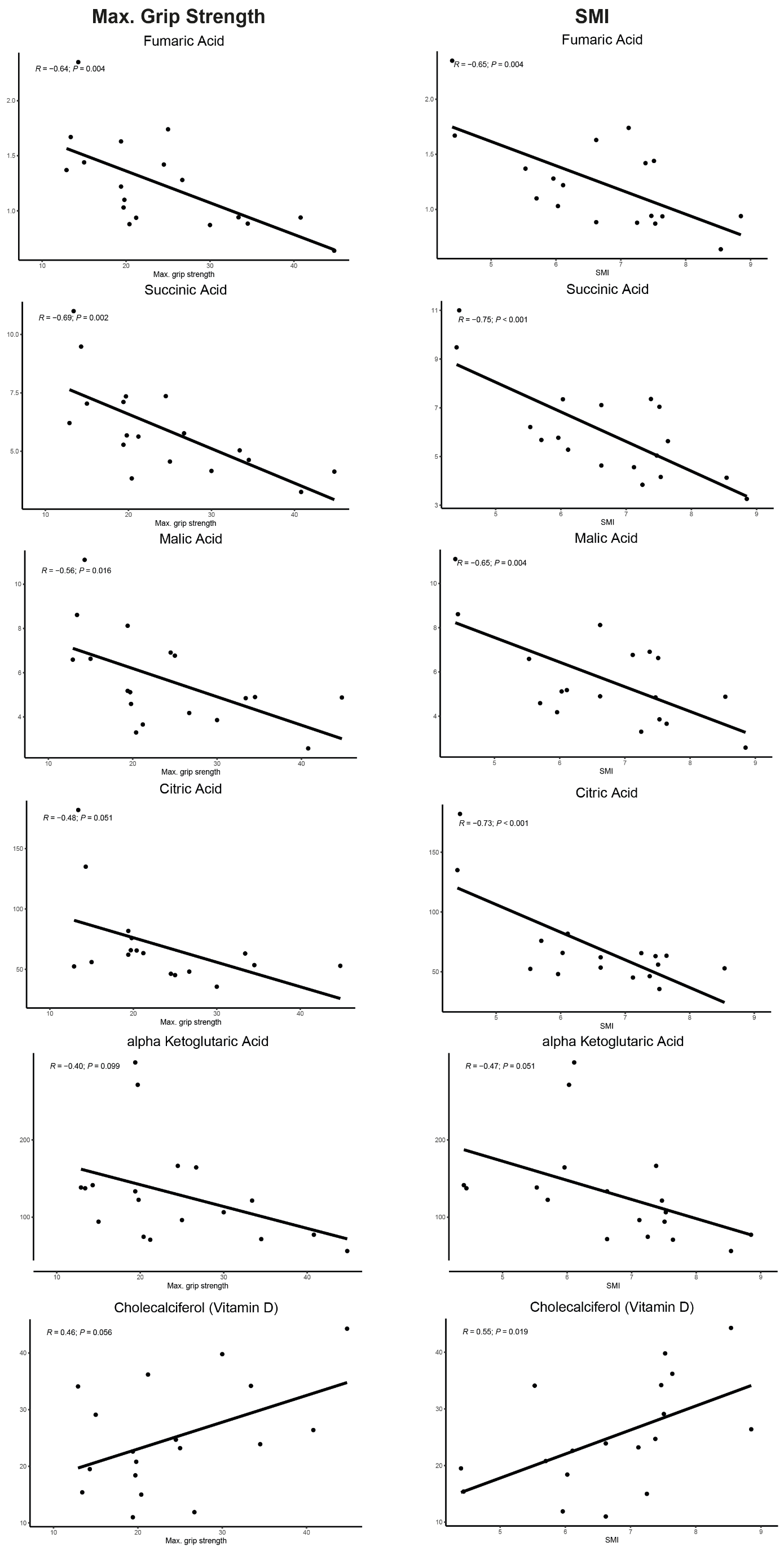


**Max. Grip Strength SMI**


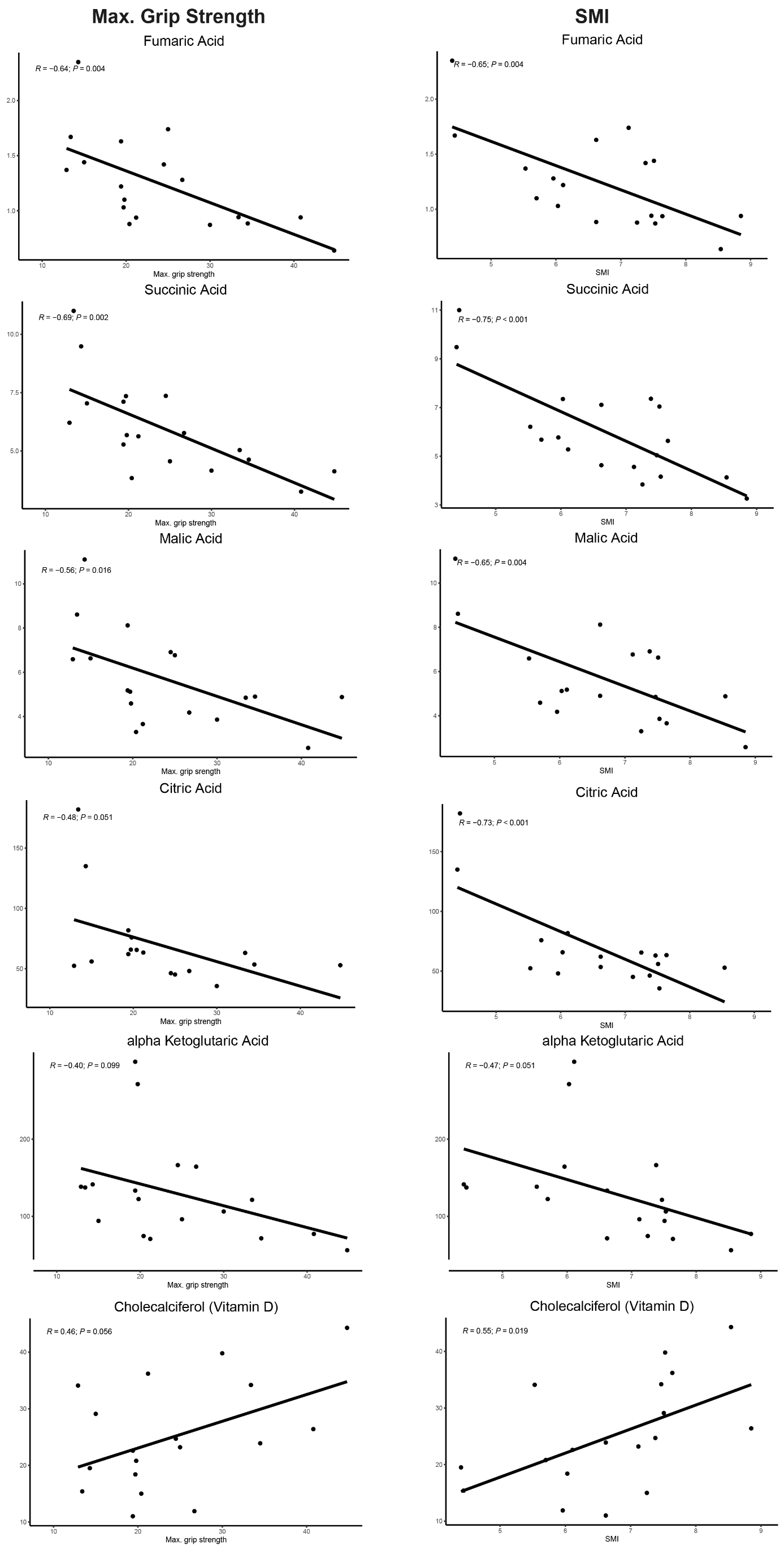


**Figure S2:** Serum analysis. The concentration of fumaric acid, succinic acid, malic acid, citric acid, α-ketoglutaric acid, and cholecalciferol (vitamin D_3_) measured in serum from 18 patients plotted separately against handgrip strength (max. grip strength) and skeletal muscle mass (SMI).
