# Supplementary material for: Exploration of mitochondrial defects in sarcopenic hip fracture patients
Source: Heliyon. 2022 Oct 19;8(10):e11143. doi: 10.1016/j.heliyon.2022.e11143 (PMC9593198; doi:10.1016/j.heliyon.2022.e11143)
Supplement: Figure S3 [file mmc3.docx]

**Max. Grip Strength SMI**


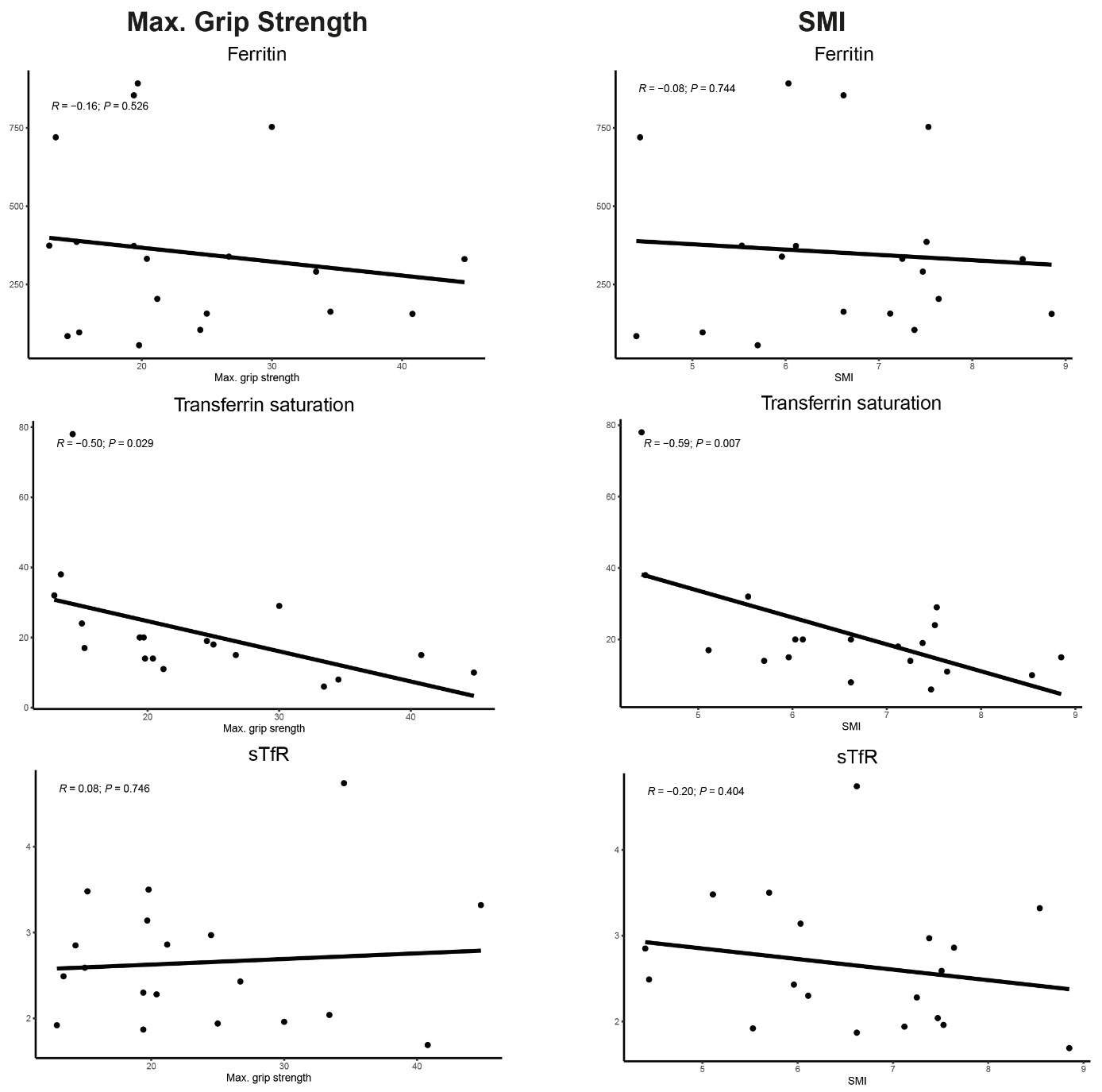
 **Figure S3:** Iron status in serum. The concentration of ferritin, transferrin saturation and the concentration of soluble transferrin receptor in a subset of 24 patients plotted separately against handgrip strength (max. grip strength) and skeletal muscle mass (SMI).
